# Collective Cell Migration Drives Morphogenesis of the Kidney Nephron

**DOI:** 10.1371/journal.pbio.1000009

**Published:** 2009-01-06

**Authors:** Aleksandr Vasilyev, Yan Liu, Sudha Mudumana, Steve Mangos, Pui-Ying Lam, Arindam Majumdar, Jinhua Zhao, Kar-Lai Poon, Igor Kondrychyn, Vladimir Korzh, Iain A Drummond

**Affiliations:** 1 Nephrology Division, Massachusetts General Hospital, Charlestown, Massachusetts, United States of America; 2 Institute for Cell and Molecular Biology, Singapore, Singapore; Wellcome Trust Sanger Institute, United Kingdom

## Abstract

Tissue organization in epithelial organs is achieved during development by the combined processes of cell differentiation and morphogenetic cell movements. In the kidney, the nephron is the functional organ unit. Each nephron is an epithelial tubule that is subdivided into discrete segments with specific transport functions. Little is known about how nephron segments are defined or how segments acquire their distinctive morphology and cell shape. Using live, in vivo cell imaging of the forming zebrafish pronephric nephron, we found that the migration of fully differentiated epithelial cells accounts for both the final position of nephron segment boundaries and the characteristic convolution of the proximal tubule. Pronephric cells maintain adherens junctions and polarized apical brush border membranes while they migrate collectively. Individual tubule cells exhibit basal membrane protrusions in the direction of movement and appear to establish transient, phosphorylated Focal Adhesion Kinase–positive adhesions to the basement membrane. Cell migration continued in the presence of camptothecin, indicating that cell division does not drive migration. Lengthening of the nephron was, however, accompanied by an increase in tubule cell number, specifically in the most distal, *ret1*-positive nephron segment. The initiation of cell migration coincided with the onset of fluid flow in the pronephros. Complete blockade of pronephric fluid flow prevented cell migration and proximal nephron convolution. Selective blockade of proximal, filtration-driven fluid flow shifted the position of tubule convolution distally and revealed a role for cilia-driven fluid flow in persistent migration of distal nephron cells. We conclude that nephron morphogenesis is driven by fluid flow–dependent, collective epithelial cell migration within the confines of the tubule basement membrane. Our results establish intimate links between nephron function, fluid flow, and morphogenesis.

## Author Summary

The kidney's job is to maintain blood ion and metabolite concentrations in a narrow range that supports the function of all other organs. Blood is filtered and essential solutes are recovered in a structure called the nephron. Human kidneys have one million nephrons, while simpler kidneys like the zebrafish larval kidney have only two. Nephrons are segmented epithelial tubules; each segment takes on a particular shape (such as convoluted, straight, or U-shaped) and plays a specific role in recovering filtered solutes. How the nephron is proportioned into segments and how some tubule segments become convoluted is not known. This work takes advantage of the simple zebrafish kidney to image living cells during nephron formation. Unexpectedly, we found that nephron cells are actively migrating “upstream” toward the filtering end of the nephron. The cells remain connected to each other and migrate as an intact tube. This is similar to a process called “collective cell migration.” We find that collective cell migration establishes the final position of nephron segment boundaries and drives convolution of the tubule. We also find that cell migration is dependent on fluid flow in the tubules, supporting the idea that organ function is important in defining its final form.

## Abstract 3

Epithelial cell shape, tubule convolution, and segment boundary position along the kidney nephron unexpectedly involve the migration of fully differentiated epithelial cells against the flow of lumenal fluid.

## Introduction

Organs form by a sequential process of cell type–specific differentiation and subsequent morphogenetic cell rearrangements. Examples of cell rearrangement and motility during early development include the movement of groups of epithelial cells during gastrulation that is coupled to the formation of the germ layers [1], and convergent extension cell movements that drive elongation of the embryonic axis [2]. Cell rearrangements during organ morphogenesis are observed in the forming heart, where bilateral groups of specified cells migrate to the embryo midline and fuse to form the primitive heart tube [3]. Epithelial organs including the kidney, lung, and liver undergo a process of branching morphogenesis where an out-pouching growth from an epithelial tube is reiteratively subdivided to form ductal organ systems [4]. In several instances of morphogenesis, epithelial cells move as intact clusters, sheets, or tubes, in a process termed “collective cell migration” [5]. This type of cell migration is observed in the caudal migration of lateral line primordia in zebrafish [6], border cell migration in *Drosophila* ovaries [7], dorsal closure in *Drosophila* embryos [8], wound healing in epithelial monolayers [9], and recently, in an in vitro model of mammary gland branching morphogenesis [10]. Collective cell migration is characterized by the movement of epithelial cells while still connected by apical cell–cell junctions and the maintenance of the polarized epithelial cell phenotype [5]. Collective cell migration can be directed by leading edge cells that extend actin-rich lamellipodial extensions in the direction of movement and transmit directional information to following cells by way of cell–cell adhesions [11]. While much is known about individual, mesenchymal cell migration, the mechanics of collective cell migration and the extent to which this process underlies organ morphogenesis are not fully known.

Morphogenesis of the vertebrate kidney results in the formation of multiple functional units called nephrons. Each nephron contains a glomerulus, or blood filter, and a tubular component that processes filtered blood plasma [12]. The organization of each nephron into discrete tubule segments that serve specialized transport functions is essential for the efficient recovery of ions and metabolites and for tissue fluid homeostasis [12]. In the mammalian kidney, the most proximal nephron segment, the proximal tubule, is responsible for the majority of salt, metabolite, and water recovery [13]. The function of nephron segments is reflected in their morphology. The reabsorptive capacity of the proximal tubule is enhanced by maximizing cell surface area in contact with vasculature and convolution of the proximal tubule within a rich vascular bed [13]. Tubule segment dysfunction underlies several human kidney pathologies including Fanconi syndrome (Online Mendelian Inheritance in Man (OMIM; http://www.ncbi.nlm.nih.gov/omim/) accession code 134600), renal tubular acidosis (OMIM 179830), and renal tubular dysgenesis (OMIM 267430). Despite the importance of nephron morphogenesis and tubule segment patterning to kidney function, little is known about how nephron segment boundaries are defined during development or what morphogenetic events drive tubule convolution.

The zebrafish pronephros is a simplified model for in vivo studies of kidney morphogenesis. Similar to the mammalian nephron, the zebrafish pronephric nephron is a segmented tubule [14–18]. Initial definition of nephron segments and kidney cell type differentiation has been linked to retinoic acid signaling and to Notch/Jagged2 interactions [15,18]. During maturation of zebrafish pronephros, nephron segment boundaries shift in a proximal direction. For example, segments defined by markers of the late proximal tubule are located in the mid-nephron shortly after kidney epithelial differentiation but are found adjacent to the glomerulus in older embryos [18]. Over the same time period, the pronephric nephron becomes convoluted in its proximal segment which later becomes closely associated with the cardinal veins [19]. Currently, nothing is known about the mechanism of these morphogenetic events.

We present here evidence that collective cell migration of differentiated pronephric epithelial cells accounts for both the proximal shift in nephron segment boundaries and proximal tubule convolution. We find that fully polarized epithelial cells initiate a concerted, proximal-directed cell migration that occurs within the confines of the tubular basement membrane. Pronephric epithelial cell migration in turn depends on lumenal fluid flow, thus linking kidney morphogenesis to kidney function.

## Results

### Proximal Displacement of Nephron Segment Boundaries and Tubule Convolution

**Figure 1:**
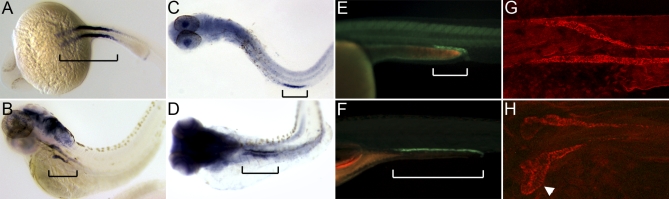
Proximal Displacement of Nephron Segment Boundaries and Convolution of the Proximal Nephron (A and B) *nbc1* in situ hybridization at 24 hpf (A) and at 72 hpf (B). (C and D) *trpM7* in situ staining at 24 hpf (C, yolk is stripped) and at 72 hpf (D). (E and F) *ret*:GFP-positive domain in live zebrafish at 30 hpf (E) and at 6 dpf (F). In (A–F), brackets show the pronephric *nbc1-*, *trpM7*-, and *ret-*positive segments. (G and H) Immunofluorescence staining for alpha1A4 subunit of the NaK ATPase at 24 hpf (G) and at 72 hpf (H).

Examination of the zebrafish pronephros at 24 and 72 hours post-fertilization (hpf) revealed a proximal-directed shift in cells expressing markers of specific nephron segments (Figure 1). The proximal tubule, identified by expression of the sodium bicarbonate co-transporter *nbc1*, spanned more than half of the total nephron length at 24 hpf (Figure 1A) but by 72 hpf occupied only the most proximal nephron domain (Figure 1B). Similarly, the late proximal nephron segment, characterized by *trpM7* expression, occupied a domain of the nephron over the yolk extension at 24 hpf (Figure 1C), but by 72 hpf had shifted in proximal direction (Figure 1D). The most distal nephron segment, homologous the collecting duct in mammals, expresses *ret1*, which at 30 hpf, occupies the distal one-fifth of the nephron (Figure 1E). The proximal boundary of *ret1* expression was dramatically shifted in a proximal direction by 6 days post-fertilization (dpf), and *ret1*-expressing cells occupied about one-half the nephron length (Figure 1F). Over the same time period, the initially (at 24 hpf) straight proximal kidney segment (Figure 1G) became convoluted by 72 hpf, folding into a hairpin structure adjacent to the glomerulus (Figure 1H).

### Cell Migration Determines Nephron Segment Boundaries and Proximal Tubule Convolution

The proximal shift in nephron segment-specific gene expression could be due to a gradual re-patterning of the nephron, with induction of new gene expression in more proximal cells. Alternatively, the shift of segment boundaries could result from segment-specific proliferation or apoptosis. It is also possible that final segment boundaries could be established by a reorganization of existing nephron segment cells without changes in gene expression. To discriminate between these possibilities, we followed the behavior of individual kidney epithelial cells by time-lapse imaging in embryos expressing green fluorescent protein (GFP) under the control of nephron segment-specific promoters.

**Figure 2:**
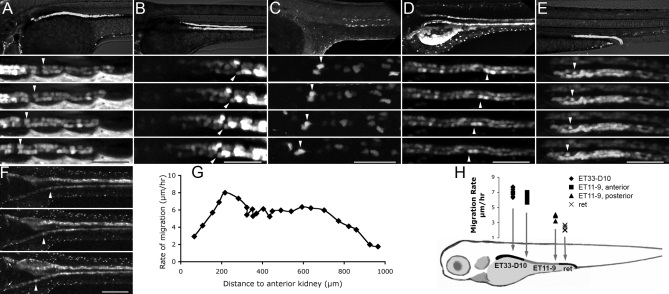
Pronephric Epithelial Cells Migrate toward the Glomerulus Individual frames of confocal fluorescent time-lapse videos at 2.5-h intervals are presented for five different kidney GFP transgenics. White arrowheads in (A–F) mark individual cells at different time points in migration. (A) ET33-D10 GFP transgenic (proximal segment).(B) ET11–9 GFP transgenic (mid segment). (C) CD41:GFP transgenic, multiciliated cells, mid kidney. (D) NaK ATPase:GFP transgenic, all transporting epithelia. (E) *ret1*:GFP transgenic, distal collecting segment. (F) NaK ATPase:GFP transgenic. Small arrows mark the convolutions forming in the proximal tubules. (G) The rate of migration in (F) is plotted as a function of the distance from proximal-most kidney. (H) Rates of migration determined for various segments of the kidney. The upper panels in (A), (B), (D), and (E) show live GFP transgenics. The upper panel in (C) shows immunofluorescent image using anti-GFP tagged antibody. The scale bar lengths are as follows: (A), (B), (D), (E): 80 μm, (C): 60 μm, (F): 200 μm. Inter-frame time interval is 2.5 h in (A–E) and 10 h in (F). The rate of migration is measured between 2 and 2.5 dpf in (G) and between 2.5 and 3 dpf in (H).

Using transgenic lines that express GFP in specific pronephric segments (Figure 2), we tracked individual GFP-expressing cells and neighboring dark cells at segment boundaries and determined whether or not changes in GFP expression (from segment-specific promoters) occurred during the period of observation. For each transgenic line, fluorescent cells at segment boundaries at the start of the time lapses remained fluorescent and, conversely, neighboring dark cells did not start expressing GFP during the time period between 36 and 96 hpf (Figure 2A–2E). These results indicate that re-patterning of pronephric epithelial cells by new induction of segment-specific gene expression is not the principle mechanism underlying the shift in nephron segment boundaries (Figure 1). Further, we did not observe apoptosis in time lapse images or by acridine orange staining (unpublished data), indicating that nephron segment shift was not due to cell death. Instead, we found that after nephron segments were specified, pronephric epithelial cells engaged in a concerted proximal-directed cell migration toward the glomerulus (Figure 2) and that this cell migration was sufficient to account for the displacement of nephron segment boundaries and proximal tubule convolution.

We imaged live pronephric cells in five different GFP transgenic lines that mark populations of epithelial cells in different nephron segments (Figure 2). The ET33-D10 line expresses GFP in the proximal nephron segment (Figure 2A and Video S1), ET11–9 expresses GFP in the mid proximal to distal segments (Figure 2B and Video S2), CD41:GFP expresses GFP in multiciliated cells (Figure 2C, Video S3, and Figure S1), NaK ATPase:GFP expresses GFP in transporting epithelia but is excluded from intermixed multiciliated cells (Figure 2D and Video S4), and the zcs/ret1 line expresses GFP in the most distal segment analogous to the collecting system (Figure 2E and Video S5) [20]. Individual cells in all five transgenic lines demonstrated migratory behavior toward the anterior or proximal nephron. Still frames of time-lapse movies from each transgenic line show displacement of individual cells (tracked with white arrowheads; Figure 2) toward the anterior. Long-term imaging of the NaK ATPase:GFP line over a period of 22 h (Figure 2F and Video S6) demonstrated that cells move continuously over the time period of 2–3 dpf and accumulate in the proximal tubule near the glomerulus (Figure 2F, small arrows).

The rate of cell migration exhibited a biphasic dependence on the position of the migrating cell along the length of the nephron (Figure 2G). Migration was slowest posteriorly, in the *ret1*-positive distal segment near the cloaca. It increased and plateaued in more proximal segments (ET11–9– and ET33-D10–positive segments) and dropped near the glomerulus (Figure 2G and 2H). This distribution of the migration rates resulted in the “piling up” of migrating cells anteriorly, as fast-moving cells decelerated near the glomerulus while more cells continued to arrive. Both ends of the pronephros remain fixed in position; the proximal end is fixed at the glomerulus that remains stationary ventral to somites 2–3 while the distal end is anchored at the cloaca [19]. The continuous arrival of migrating cells at the fixed proximal end of the pronephros resulted in folding of the proximal pronephric tubule into a hairpin-like structure, the proximal convolution (Figure 2F and Videos S6 and S7). The most distal, *ret1*-expressing segment, in contrast, became long and straight. This distal segment spanned as much as half of the nephron length by 6 dpf (Figure 1F).

### Pronephric Cells Move by a Process of Collective Cell Migration

**Figure 3:**
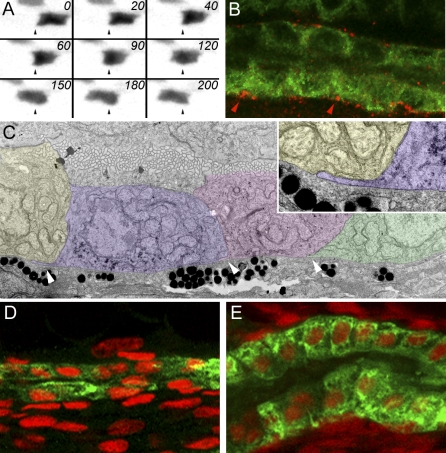
Pronephric Epithelial Cell Migration (A) Time-lapse images of an individual pronephric multiciliated cell in a 2.5 dpf CD41:GFP transgenic fish at 20-min intervals. Dynamic transient cytoplasmic projections are seen from the basal cell surface, most visible in frames 0 min, 60 min, and 120 min. (B) Double immunofluorescence staining of the pronephros in 3-dpf fish with anti-phospho-FAK antibody (red) and anti-alpha6 NaK ATPase (green). Phospho-FAK staining is positive at the epithelial cell interfaces along the basement membrane. (C) Transmission electron microscope images show that migrating epithelial cells send cryptic lamellipodia along the basement membrane in the direction of migration (arrowheads). Here three migrating cells are false colored to distinguish individual cells. Inset shows a single basal cytoplasmic projection. (D and E) The change in epithelial morphology of the proximal kidney (ET33-D10 GFP segment) between 36 hpf (D) and 96 hpf (E). At 36 hpf, the proximal epithelial cells are low cuboidal and the tubular diameter is small. At 96 hpf, the proximal tubule has much larger diameter and the epithelial cells become columnar. The ET33-D10 transgenic embryos were stained with anti-GFP antibody (green Alexa 488 secondary) and DAPI (red pseudocolor).

The concerted movement of tubule cells relative to other tissue implied that individual epithelial cells would be polarized in the direction of movement, would show protrusive membrane activity, and would exert traction on the tubule basement membrane for forward movement. We examined single cell behavior in time-lapse videos to test whether pronephric cells exhibited any of these behaviors. An example of an individual CD41:GFP-positive cell imaged every 20 min is shown in Figure 3A and Video S8. Migrating cells extend basal membrane protrusions in the direction of forward movement and appear to make transient contacts with the tubule basement membrane (Figure 3A). Membrane domains engaged in dynamic reorganization of focal adhesions are often positive for phosphorylated Focal Adhesion Kinase (phospho-FAK). Basal surfaces of tubule segments exhibiting movement were positive for phospho-FAK by antibody staining (Figure 3B), further supporting the idea that basal cell protrusions were associated with adhesion to the tubule basement membrane. The migration of epithelial cells often involves a transition to a more mesenchymal phenotype and loss of cell–cell adhesion [21]. We examined migrating cells by electron microscopy and found instead that the pronephric epithelial cells remained fully polarized with brush border membranes and joined together by adherens junctions (Figure 3C). Interestingly, we also observed that a majority of cells extended lamellipodia along the tubule basement membrane at their forward edge and under the cell in front (Figure 3C; white arrowheads and inset). In electron microscopy of one complete tubule, 21 out of 32 basal cell junctions studied (66%) showed forward-directed basal lamellipodia. Thus, tubule epithelial cells maintain apical connections to their neighbors in front and behind while exhibiting free movement of their basal surfaces, engaging in a form of collective cell migration.

### Collective Cell Migration Is Coupled to Cell Proliferation in the Distal Nephron

Since both the distal and proximal ends of the pronephros are fixed in position at the cloaca and glomerulus, respectively, the proximal cell migration we observe would be expected to cause an overall lengthening the pronephros while depleting the number of cells in distal segments. Measurements of the pronephros did reveal a steady lengthening of the tubule over the period of observation (1,264 ± 15 μm at 48 hpf (*n* = 9 tubules); 1,564 ± 25 μm at 96 hpf (*n* = 7 tubules)) however we did not observe a significant “thinning” of cells in the distal segment. We therefore asked whether the lengthening of the distal segment was accompanied by an increase in the number of cells in that segment. We examined the total number of DAPI-stained nuclei in the distal, *ret1*:GFP-positive segment, comparing embryos at 36 hpf and at 4 dpf. The number of *ret1*:GFP cells doubled over this time period (207 nuclei at 36 hpf to 411 at 4 dpf). This increase in cell number was proportional to the increase in length of the *ret1*-GFP positive segment (from 286 μm to 570 μm). In contrast, the number of proximal, ET33-D10-GFP–positive cells did not change significantly over the same time window (208 nuclei at 36 hpf to 242 nuclei at 4 dpf), whereas the length of the GFP-positive domain shortened from 662 μm to 300 μm. This compression of the proximal segment without a change in cell number is consistent with the transition from a cuboidal to columnar cell morphology (Figure 3D and 3E). To test whether disproportionate cell proliferation in the distal nephron segment played a role in stimulating forward migration by “pushing” cells proximally, we imaged tubules in embryos incubated in the presence of 60 μM camptothecin to block cell proliferation. Short-term (<8 h) treatment of the embryos with camptothecin had no effect on the migration rate of the pronephric epithelial cells (Video S9). Camptothecin incubation of a similar duration arrested early embryo development (Figure S2C and S2D) and blocked BrdU incorporation (Figure S2A and S2B), indicating that it effectively blocked cell division. We conclude the following: (1) proximal-directed cell migration is not driven by distal cell proliferation, but (2) the lengthening of the distal nephron segment is compensated by cell proliferation and (3) compression of the proximal tubule occurs without significant cell proliferation in this segment.

### Initiation of Cell Migration Correlates with Onset of Tubule Fluid Flow

**Figure 4:**
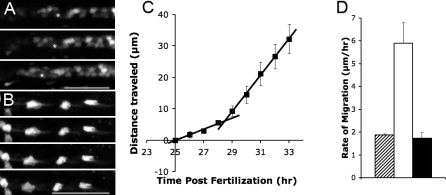
Timing of Pronephric Cell Migration (A) NaK ATPase:GFP transgenic showing initiation of cell migration after 29 hpf. Frame interval: 4.5 hr, scale bar: 70 μm. (B) Time-lapse of the CD41:GFP transgenic 5-dpf embryo showing cessation of migration. Frame interval: 2.5 hr, scale bar: 60μm. (C) A sharp increase in the distance traveled occurs at 28.5 hpf. The two regression lines intersect at 28.5 hpf. Each point represents distance traveled by epithelial cells between 25 hpf and a given time point. The data plotted represent averages from three different embryos (D) Comparative rates of epithelial migration before 28.5 hpf (striped bar), after 28.5hpf (white bar), and after 5 dpf (black bar).

In our time-lapse studies we observed that prior to 24 hpf or after 4 dpf, epithelial cells migrated at a slow rate (<2 μm/h, Figure 4B and 4D). In contrast, between 36 hpf and 4 dpf, the migration rate was markedly increased (6–8 μm/h; Figure 2). To determine when the transition between the slow migratory and fast migratory phenotype occurred, we imaged tubules starting around 24 hpf. In three independent time-lapse studies, we observed a reproducible sharp increase in cell migration rate at 28.5 hpf from 2 μm/h to 6 μm/h (Figure 4A, 4C, and 4D, and Video S10). Interestingly, this increase in motility was preceded by the onset of active fluid transport into the pronephros. Fluid transport into the pronephric nephron lumen could be detected by mechanically obstructing the pronephros at 24 hpf, prior to the formation of the glomerulus, and observing rapid dilation of the pronephric tubules and the formation of proximal tubule cysts within one hour (unpublished data). Fluid output at the cloaca could also be detected by covering the posterior yolk extension of an unobstructed 30hpf embryo with petroleum jelly and mineral oil and imaging pronephric excretion form fluid droplets at the outside surface of the cloaca (Video S11). Since fluid accumulation in the pronephros occurred before the onset of glomerular filtration [19], fluid input to the tubule lumen must occur by active solute transport across the pronephric epithelium [22].

### Nephron Fluid Flow Is Required for Proximal Tubule Convolution and Cell Migration

**Figure 5:**
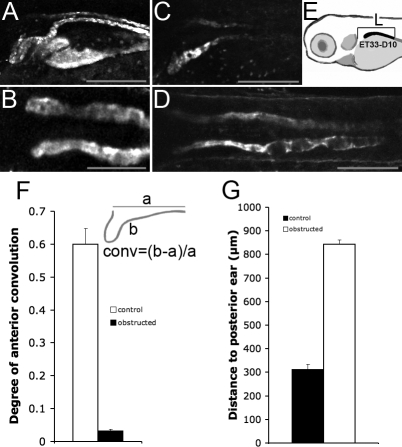
Pronephric Obstruction Prevents Convolution and Proximal Progression of the Proximal Tubule Segment (A) The proximal tubule in NaK ATPase:GFP transgenic larvae is folded into a hairpin structure at 4 dpf. This anterior convolution is abolished in kidneys obstructed at 24 hpf (B). (C and D) The ET33-D10 GFP positive nephron segment (C) fails to progress anteriorly in 84hpf obstructed kidneys (D). (E) method of measurement. (F) Convolution was defined as the ratio of the length of the anterior portion of the pronephros in NaK ATPase:GFP transgenics minus the length of a straight line connecting the ends of this segment divided by the length of this straight line. The anterior segment was arbitrarily defined as that anterior to yolk extension (conv = (b – a)/a, as shown in the inset). The white bar is the control condition (*n* = 10). The black bar indicates the obstructed kidney (*n* = 19). Obstruction was induced at 24 hpf, measurements were performed at 96 hpf. (G) Measurement of the proximal segment progression. The black bar indicates the control nephrons (*n* = 13). The white bar indicates obstructed nephrons (*n* = 17). Obstruction was induced at 24 hpf. The measurement was performed at 84 hpf.

**Figure 6:**
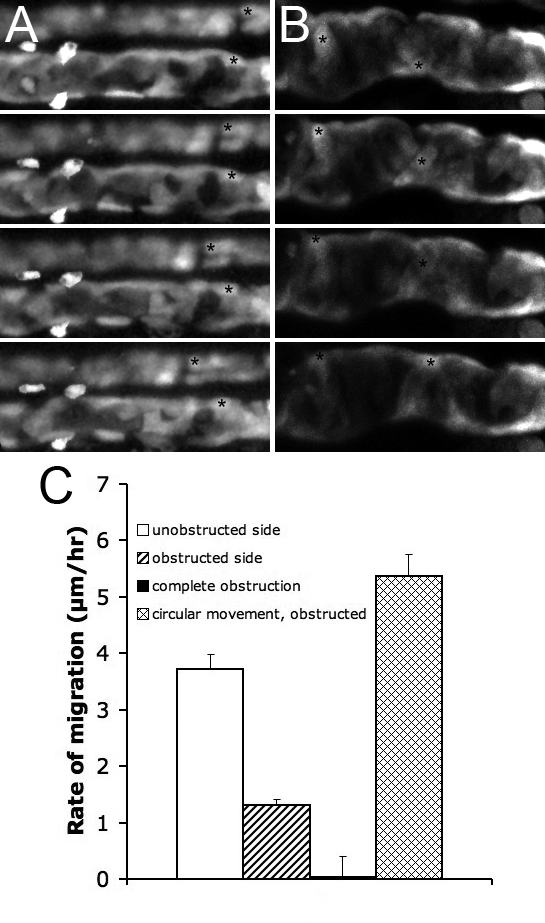
Pronephric Obstruction Blocks Proximal Cell Migration (A) Unilateral obstruction, NaK ATPase:GFP, the obstructed side is shown below the control side. (B) Complete obstruction, ET11–9 GFP transgenic. The epithelial cells move circumferentially. Frame intervals in (A, B) are 2.5 h, scale bar: 70 μm. (C) Rates of migration in various conditions, from left to right: unilateral obstruction, unobstructed side (white bar); unilateral obstruction, obstructed side (striped bar); complete obstruction, rate of anterior migration (black bar); complete obstruction, rate of circumferential movement (mesh bar). Each bar represents average over a number of cells within a single experiment.

The correlation between onset of cell migration and initiation of nephron fluid flow suggested that the fluid flow could act as an initiating signal for proximal cell migration. All nephron fluid flow can be eliminated by simple mechanical obstruction of the distal nephron [23]. Remarkably, distal nephron obstruction effectively abolished convolution of the proximal tubule (Figure 5) and proximal cell migration (Figure 6). In 4-dpf NaK ATPase:GFP transgenic larvae, the proximal tubule has a hook-like structure in control larvae (Figure 5A), whereas in obstructed larvae, the proximal tubule remained straight with no sign of convolution (Figure 5B). Similar morphological results were observed in the proximal tubule-specific transgenic line ET33-D10 (Figure 5C and 5D). The quantitation of these results in a larger number of NaK ATPase:GFP-transgenic embryos confirmed that convolution was effectively abolished by nephron obstruction (Figure 5F). We also examined whether distal obstruction would block progression of the proximal nephron segment boundary toward the glomerulus. The distance between the posterior border of the ear and the posterior edge of the ET33-D10 GFP-positive segment served as a measure of segment migration (Figure 5E). Complete obstruction blocked the shortening and convolution of the proximal segment (Figure 5C, 5D, and 5G; see also Figure 1A and 1B). Time-lapse imaging of tubule cells in obstructed larvae confirmed that the lack of tubule convolution and proximal progression was due to lack of proximal cell migration (Figure 6A and 6B). In unilaterally obstructed larvae, migration was blocked specifically on the obstructed side while proximal migration continued on the unobstructed side (Figure 6A and Video S12). Interestingly, while obstruction blocked proximal cell migration, in several instances pronephric cells continued to move circumferentially such that the tubule appeared to rotate on the lumenal axis (Figure 6B and Video S13). Quantitation of migration rates of multiple cells revealed that obstruction severely reduced or abolished proximal migration. However, the rate of circumferential cell motility was similar to the rate of proximal cell migration in control larvae (Figure 6B and 6C).

**Figure 7:**
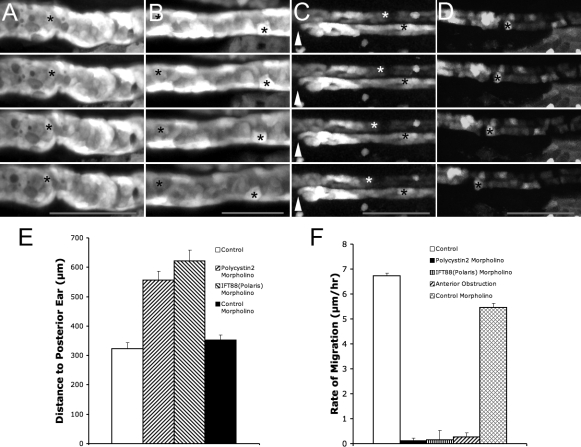
Decreasing Pronephric Fluid Flow Suppresses Anterior Migration (A–D) Time-lapse frames of ET33-D10 GFP transgenics, 2.5 dpf. Time interval between frames is 2.5 h, scale bar: 80 μm (in bottom frame). (A) *polycystin2* morphant embryo; asterisk marks stationary cell in the pronephros. (B) *ift88* (*polaris*) morphant embryo; asterisks mark stationary cells in the pronephros. (C) Anterior (proximal) obstruction; arrowhead marks position of unilateral anterior obstruction; white asterisk marks a migrating cell in the unobstructed nephron; black asterisk marks stationary cell in the obstructed nephron. (D) Control morpholino injected embryo showing normal rate of proximal cell migration (asterisk marks a migrating cell). (E) Measurement of proximal nephron segment shortening in ET33-D10 GFP transgenics (shorter lengths indicate further proximal progression as described in Figure 5E). From left to right: Control uninjected embryos (*n* = 36); *polycystin2* morphant embryos (*n* = 20); *ift88* (*polaris*) morphant embryos (*n* = 10); Control morpholino injected embryos (*n* = 17) (F) Rates of proximal cell migration, from left to right: Control; *polycystin2* morphant embryo; *ift88* (*polaris*) morphant embryo; anterior obstruction; Control morpholino.

We and others have shown that morpholino knockdown of the transient receptor potential (TRP) channel *polycystin2* results in constriction of the distal nephron lumen, reduced fluid output from the pronephros, and proximal tubule cyst formation [24]. In cystic *polycystin2* knockdown embryos, proximal cell migration was completely arrested (Figure 7A and 7E), which is consistent with the lack of cell migration due to nephron obstruction. However, similar to the obstructed larvae, on occasion we observed persistent circumferential movement of cells, indicating that *polycystin2* loss of function did not inhibit cell motility in general (Video S14). Similar to *polycystin2* knockdown, interfering with cilia assembly by knockdown of intraflagellar transport protein (IFT) expression results in functional obstruction of the nephron due to loss of fluid propulsion with consequent tubule dilatation and proximal cyst formation [23]. Knockdown of *ift88* using antisense morpholino oligos arrested proximal migration of nephron epithelial cells similar to that seen in mechanically obstructed and *polycystin2* knockdown nephrons (Figure 7B and 7E, Video S15). Consistent with inhibition of cell migration, both *polycystin2* and *ift88* knockdown resulted in failed shortening and lack of convolution of the ET33-D10 GFP-positive proximal nephron segment (Figure 7D). During this stage of development, the two pronephric glomerular primordia are also moving toward the embryo midline to eventually fuse and form the glomerulus. Neither disrupting expression of *polycystin2* or *ift88*, nor inducing physical obstruction of the pronephros, prevents glomerular fusion (see Figure S3 and [23,24]). These data suggest that glomerular morphogenesis occurs by a mechanism that is distinct from the mechanisms driving proximal cell migration.

Both mechanical obstruction and knockdown of *polycystin2* or *ift88* result in cystic dilation of the pronephros [23,24], raising the possibility that cell migration could be affected by cell stretch. To assay cell migration independently of tubule dilatation and cell stretching, we induced a proximal obstruction by transecting the nephron at the proximal edge of the yolk extension. This would be expected to eliminate fluid input to the distal (post-obstruction) tubule without causing tubule dilatation. Proximal obstruction completely eliminated cell migration in the remaining tubule segment distal to the obstruction, whereas cells in the contralateral non-obstructed tubule continued to migrate (Figure 7C and 7E). These data indicate that nephron fluid flow is essential for directed migration and that lumenal distension did not play a role in arresting proximal migration.

**Figure 8:**
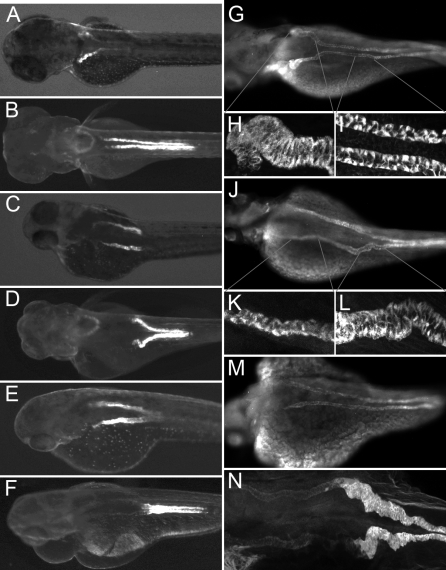
Eliminating Glomerular Filtration Results in Ectopic Tubule Convolution All embryos were imaged at 84 hpf. (A and B) Control condition, ET33-D10 transgenics (A) and ET11–9 transgenics (B). (C and D) Cardiac *tnnt2* morpholino injected embryos, ET33-D10 transgenic (C) and ET11–9 transgenic (D). (E and F) Cardiac *tnnt2* and *ift88* (*polaris*) morpholino injected embryos, ET33d10 transgenic (E) and ET11–9 transgenic (F). (G–M) Anti-alpha6F NaK ATPase stained embryos. (G) Control embryo. (H and I) Close-ups of the proximal and mid segments. (J) Cardiac *tnnt2* morpholino injected embryo. (K and L) Close-ups of the proximal and the mid segments. (M) Cardiac *tnnt2* and *ift88*(*polaris*) morpholino-injected embryo. (N) ET11–9 GFP transgenic embryo injected with cardiac *tnnt2* morpholino, stained with anti-alpha6 NaK ATPase (faint), and anti-GFP (bright).

Fluid flow in the zebrafish pronephros can be generated by at least two mechanisms: fluid input at the proximal nephron by glomerular filtration, and propulsion of fluid more distally by lumenal cilia bundles acting as local “fluid pumps” [23]. Fluid input by glomerular filtration is driven by blood pressure. We eliminated glomerular filtration by arresting heartbeat using an antisense morpholino against cardiac troponin T (*tnnt2/silent heart*; [25]). The absence of glomerular filtration markedly inhibited collective cell migration and nephron convolution in the proximal, ET33-D10 GFP-positive nephron segment (Figure 8C and Video S16) compared to control (Figure 8A and Video S1). While cell migration was blocked proximally, it persisted in the more distal ET11–9 GFP-positive nephron segment (Video S17). The proximal end of the ET11–9 segment showed the most significant decrease in the migration rate, which dropped from 5.5 μm/h to 2.5 μm/h across the anterior (proximal) 100 μm of the ET11–9 GFP-positive segment. Strikingly, elimination of glomerular filtration shifted the position of nephron convolution distally to an ectopic position at the boundary of the ET33-D10 and ET11–9 tubule segments (Figure 8C, 8D, and 8N), suggesting that convolution occurs at a point where further proximal cell migration is blocked. In addition, epithelial morphology was significantly changed in *tnnt2* morphants compared with control embryos at 84 hpf. In controls, the most proximal, convoluted segment showed a distended tubule filled with columnar epithelial cells (Figure 8G and 8H; also Figure 3) whereas more distal segments were not distended and contained cuboidal epithelial cells (Figure 8G and 8I). In *tnnt2* morphants, this pattern was reversed and the distended portion of the tubule was found at the position of ectopic convolution—at the boundary of ET33-D10 and ET11–9 segments (Figure 8J, 8L, and 8N)—and was filled with columnar cells, whereas the proximal tubule was not distended and contained cuboidal epithelial cells (Figure 8J, 8K, and 8N). This boundary between the ET33-D10 and ET11–9 tubules also roughly marks the most proximal extent of the CD41:GFP-positive multiciliated cell population (Figure 2 and Figure S4). To test whether persistent migration of the more distal cells in *tnnt2* morphants was dependent on cilia-driven fluid flow, we disrupted cilia function with an *ift88* (*polaris*) antisense morpholino [23]. Importantly, when combined with knockdown of *tnnt2*, *ift88* knockdown eliminated the ectopic tubule convolution seen in *tnnt2* morphants (Figure 8E, 8F, and 8M). The results show that lumenal fluid flow is essential for proximal-directed, collective cell migration and that tubule convolution occurs specifically at the point of resistance to further cell movement.

## Discussion

Morphogenesis and segmentation of the kidney nephron underlies its essential function in the processing of filtered blood and the recovery of ions and metabolites. We have shown that nephron tubule patterning and morphology is controlled by a process of collective cell migration where differentiated, polarized kidney epithelial cells move en masse in a proximal direction toward the glomerulus. Our results also show that the initial definition of nephron segments associated with the expression of distinct transgenic markers and sets of ion transporter genes [18] is complete early in nephron formation and that the ultimate position of nephron segment boundaries and tubule convolution is a function of tubule cell migration. Kidney tubule cell migration is, in turn, controlled by fluid flow within the nephron, demonstrating an essential relationship between organ morphogenesis and organ function.

### Collective Cell Migration and Kidney Organogenesis

Collective cell migration describes the coherent movement of cell groups, while the cells themselves remain connected by cell–cell junctions and maintain constant positions while migrating [5]. Examples include the caudal migration of clusters of lateral line progenitors in zebrafish [6], border cell migration on *Drosophila* oocytes [7], and wound healing in cultured epithelial cell monolayers [9]. In each of these cases, epithelial cells adhere to each other by apical adherens junctions, while their basal surfaces are free to engage in dynamic, directed lamellipodial extensions associated with matrix adhesion. In wounded monolayers, basal lamellipodia are seen not only in leading edge cells but also in cells behind the edge, indicating that these cells are also actively involved in maintaining the migration of the monolayer [26]. Similarly, we found that kidney epithelial cells along the entire mid-portion of the nephron maintain cell–cell attachments while they extend basal lamellipodia in the direction of migration. This suggests that all tubule cells in this segment are actively involved in proximal migration as opposed to being passively “pulled along” by a smaller group of actively motile leading cells. Alternatively, it remains possible that a subset of proximal cells convey directional information to more distal cells and organize their proximal-directed migration by way of mechanical forces applied to cell–cell contacts [11]. Unlike the case of migrating lateral line primordia and wound healing in monolayers, in the pronephros, there is no free leading edge to the epithelial cell group that directs other cells to fill a gap or area of open matrix. Instead, all kidney tubule cells migrate proximally and compress cells at the leading edge into a columnar morphology, distorting tubule shape and creating the characteristic convolutions of the proximal tubule. Convolution of the proximal tubule also occurs in the mammalian kidney, where proximal tubule cells appear compressed and are more columnar in shape whereas in the more distal nephron, the cells are low cuboidal in shape [13]. The similarities in cell morphology between mammalian and zebrafish nephrons raise the intriguing possibility that collective cell migration of tubular epithelial cells may be a general feature of vertebrate kidney morphogenesis.

### Fluid Shear Forces and Organogenesis

Collective cell migration in the zebrafish pronephros required lumenal fluid flow, suggesting that fluid shear stress in the tubule lumen may be essential for nephron morphogenesis. Similar arguments have been made for biomechanical effects of fluid shear forces on cardiac morphogenesis. Physical obstruction of the cardiac inflow tract results in heart chamber malformation, decreased heart looping, and atrioventricular (AV) valve malformation [27]. The absence of endocardial cushions in blocked hearts lead Hove et al. to suggest that fluid shear stress was essential for the early steps in cardiac valve formation [27]. Subsequent studies using pharmacological inhibition of heart function have suggested an alternative mechanism of endocardial cushion formation that involves myocardial contractions per se, independent of shear forces [28]. We have previously presented evidence implicating fluid shear force in morphogenesis of the glomerulus in zebrafish [29]. Mutants lacking blood flow fail to express MMP2 in endothelial cells and do not invade and remodel glomerular podocytes [29].

Perhaps the best-studied example of cellular response to fluid shear stress is the response of endothelial cells to blood flow. Endothelial cells sense and respond to fluid shear using a system of adhesion molecules including PECAM and VE-cadherin, integrin activation, activation of receptor tyrosine kinases, calcium influx, and modulation of the cytoskeleton by Rho family GTPases [30]. Fluid shear first induces lamellipodial cell extensions followed by basal protrusions and new focal adhesion formation in the direction of flow, which are controlled by polarized distribution of rac and cdc42 [31,32]. Subsequent migration requires remodeling of adhesions and release of cell substratum attachments at the rear of the migrating cell [33]. Migration of pronephric epithelial cells is likely to involve similar basic mechanisms. For instance, we observe a strong correlation between the presence of directed lamellipodial extensions of epithelial cells on the tubule basement membranes and basal phospho-FAK staining, suggesting that pronephric epithelial cells actively remodel their matrix attachments as they migrate. It is also likely that specific integrin subunits are required for pronephric cell migration, as they are in endothelial cells [34,35]. A notable difference between pronephric cell migration and endothelial cell migration is that pronephric cells migrate against flow instead of in the direction of flow. The similarities and differences between these two systems are likely to prove useful in resolving how mechanical forces establish self-perpetuating cell movement.

It is currently not clear how kidney epithelial cells might initially sense and respond to fluid flow. Manipulation of two known members of the mechanosensory TRP ion channel family, *polycystin2* and *trpM7*, did not conclusively link motility to TRP channel activity. *polycystin2* morphants have an obstructed phenotype [24], which precludes the analysis of Polycystin2 in fluid flow sensing. Also, *polycystin2* morphant tubule cells continue to move in a circumferential fashion, similar to other obstructed models, indicating that *polycystin2* is not required for motility *per se*. Knockdown of *jagged2* re-patterns the nephron and eliminates *trpM7* expression [15]; however, this did not affect the extent of tubule cell migration (unpublished data). Further studies will be required to determine whether mechanosensory channels are involved in tubule cell migration, or whether shear forces are detected by other mechanisms, as has been described in endothelial cells [36].

While the data is compelling for a role of shear stress in nephron epithelial cell migration, an alternative mechanism for stimulation of motility could involve the delivery of a chemotactic peptide to the nephron lumen by glomerular filtration or secretion that is then carried down the nephron by fluid flow. Nephron epithelial cells could internalize or otherwise inactivate such a ligand and establish a linear concentration gradient along the length of the nephron. While we cannot completely rule out this potential mechanism, aspects of our data suggest that it is unlikely. First, elimination of glomerular fluid input by blocking cardiac contractions did not prevent cell migration in distal nephron segments. This indicates that delivery of a chemotactic peptide by glomerular filtration is not required for distal cell motility. If a hypothetical chemoattractant were instead secreted by the epithelial cells themselves, the *silent heart/tnnt2* morphant data would indicate that proximal tubules do not secrete the factor, since these cells are not motile in *tnnt2* morphants. However, proximal tubule cells are highly motile in normal embryos with filtration-driven fluid flow. Also, it is difficult to imagine how a gradient of secreted chemoattractant could generate the circumferential cell movement observed in obstructed nephrons. It is more likely (but not proven) that this curious cell behavior could result from local vortex fluid forces generated by cilia beating in the tubule lumen. Indeed, we consistently observed the presence of such vortex currents in obstructed kidneys as evidenced by the movement of lumenal debris (unpublished data).

### Collective Cell Migration and Kidney Pathology

Does collective cell migration play a role in organ pathology and tissue repair? In utero obstruction of the kidney outflow that is induced experimentally or due to congenital anomalies results in kidney dysplasia that involves both cystic changes with the failure of nephrons to mature [37]. Interestingly, urogenital obstruction often results in a reduction in the ratio of proximal-to-distal tubule segments, as assayed by cell morphology in kidney biopsy specimens [37]. This phenomenon would be consistent with lack of nephron fluid flow preventing normal cell migration and proximal tubule convolution. Autosomal renal tubular dysgenesis (OMIM 267430) is a severe disorder characterized by lack of proximal tubule differentiation [38]. Low blood pressure and hypoperfusion of the fetal kidney are proposed to be common features of several conditions that lead to renal tubular dysgenesis [39]. A reduction in filtration-driven nephron fluid flow would be expected in this context, again raising the possibility that failure to stimulate fluid flow and cell migration could contribute to nephron pathology. The phenomenon of intraepithelial migration may also play a role in the adult kidney homeostasis. Kidney tubules are known to regenerate after damage from acute insult [40]. In this case, however, proliferating tubule cells undergo a mesenchymal transformation before repolarizing to replace missing epithelial cells [41]. Nonetheless, it is possible that the final re-establishment of overall tubule morphology and nephron segment boundaries after injury could involve collective tubule cell migration, similar to what we describe.

## Materials and Methods

### Zebrafish lines and embryo handling.

The NaK ATPase alpha1A4:GFP transgenic line [15], the ET(krt8:EGFP)sqet11–9 line and the ET(krt8:EGFP)sqet33-d10 line [42–44], the CD41:GFP line [45], and ret1:GFP line (zcs) [20] were raised and maintained as described in (Westfield, 1995). The ET(krt8:EGFP)sqet11–9 and ET(krt8:EGFP)sqet33-d10 lines are referred to as ET11–9 and ET33-D10. Embryos were kept at 28.5°C in E3 solution until 24 hpf and E3 solution containing 0.003% PTU (1-phenyl-2-thiourea, Sigma) after 24 hpf.

### Generation of novel ET transgenics.

To create transgenic lines ET33-D10 and ET11–9, the embryos of primary transgenic lines ET33 –D10 and ET11–9 were injected with mRNA encoding Tol2 transposase resulting in transposition of Tol2 transposon within a genome as described before and F_1_ heterozygote embryos were screened for new GFP expression patterns [42–44].

### Time lapse imaging of cell migration.

Five transgenic lines expressing GFP in the pronephros were used to image cell migration in time lapse videos: ET33-D10, ET11–9, ret1:GFP, CD-41:GFP, and NaK ATPase:GFP. Embryos from incrossed adults of each line were anesthetized using 0.2mg/ml tricaine, immobilized/oriented in 2% low melting point agarose, and mounted on the stage of a Zeiss LSM5 Pascal confocal microscope. At 20–45 minute intervals the pronephros was imaged in a z-series stack using a 40X water dipping lens. Maximum intensity projections of each stack were generated and assembled into a time lapse videos using Zeiss pascal software. Frames were additionally processed to adjust contrast using Adobe photoshop and reassembled into quicktime movies using Graphic Converter (Lemke software).

### Morpholino antisense oligonucleotides.

The following morpholino oligos were used: *ift88* (GenBank [http://www.ncbi.nlm.nih.gov/Genbank/] accession code NM_001001725) exon1d: 5′-AGCAGATGCAAAATGACTCACTGGG-3′ 0.2 mM; *polycystin2* (NM_001002310) exon12d: 5′-CAGGTGATGTTTACACTTGGAACTC-3′0.25 mM; Standard Control: 5′-CCTCTTACCTCAGTTACAATTTATA-3′ 0.25 mM; and *tnnt2* (NM_152893) ATG: 5′-CATGTTTGCTCTGATCTGACACGCA-3′ 0.125 mM.

Morpholinos were diluted in 100 mM KCl, 10 mM HEPES, 0.1% Phenol Red (Sigma) to the following concentrations: 0.2 mM *ift88*, 0.125 mM *tnnt2*, 0.25 mM *polycystin2*, 0.25 mM Standard Control. When used together, *ift88* and *tnnt2* morpholino concentrations were 0.15 and 0.125 mM, respectively. A fixed volume of 4.6 nl was injected into each embryo at 1–2 cell stage using a Nanoliter2000 microinjector (World Precision Instruments). Morpholino efficacy was confirmed by observing expected phenotypes: the *ift88* morpholino induced glomerular cysts [23], the *tnnt2* morpholino eliminated heart contractions [25], and the *polycystin2* morpholino induced a characteristic spiral dorsal axis curvature [24].

### Surgical obstruction of the pronephros.

24-hpf embryos were dechorionated and anesthetized with tricaine. An incision just anterior to the cloaca and perpendicular to the long axis of the embryo was made using a razor blade. For anterior obstruction, the incision was made at the level of yolk-to- yolk extension interface. The embryos were allowed to heal for few minutes and then were transferred into new, clean E3 egg water.

### Morphometry of GFP transgenic fish.

The ET33-D10 GFP transgenic line was used to measure the extent of proximal cell migration in groups of embryos at fixed times of development. The embryos were anesthetized using 0.2 mg/ml Tricaine and immobilized/oriented in 2% low melting point agarose. The embryos were photographed using Leica DFC 300 FX camera mounted on a Leica MZ 16F fluorescent dissecting microscope using Leica Application Suite version 2.4.0 R1 (Leica Microsystems). Images were imported into Aperio ImageScope viewer (Aperio Technologies, Inc.) and the distance from the posterior edge of the otic vesicle to the distal edge of the GFP positive pronephric domain was determined for each image. Measurements were normalized to a stage micrometer measurement. The measurement of the proximal tubule convolution was performed using NaK ATPase:GFP transgenic fish. Embryos (with or without obstruction) were anesthetized at 96 hpf, immobilized, and photographed as described above. The images were imported into NIH ImageJ software and curved line lengths were measured using polygon approximation. The ratios c = (b – a)/a were generated as shown in Figure 5F. The measurement of migration rates was performed using Zeiss Image Examiner software (Carl Zeiss, Inc.). Individual cells were traced in time-lapse images and the distances traveled were measured in relationship to arbitrary stationary reference points in the skin (skin GFP expressing cells or iridophores). Four–ten cells were traced per experiment, and the rates of migration were averaged. Morphometric results were plotted using Excel (Microsoft Corp.). Regression fitting of the data was performed using Matlab (The Mathworks, Inc.)

### Chemical treatment of the embryos and BrdU incorporation.

Camptothecin was purchased from Sigma and applied to a final concentration of 30 μM or 60 μM in presence of 1% DMSO at various stages of embryo development [46]. For time-lapse study, the embryo was mounted in agarose and preincubated in 60 μM camptothecin for 1 h prior to the start of time lapse. To test the BrdU incorporation, the embryos were incubated in 60 μM camptothecin for 2 h followed by addition of 10 mM BrdU (Sigma) and further incubated for 2 h. The embryos were then fixed in 4% PFA, washed with PBST (0.1% Tween 20 in PBS) and treated with 10 μg/ml proteinase K for 30 min. The embryos were washed again and treated with 2N HCl for 1 h. The embryos were then prepared for the whole-mount immunohistochemistry using anti-BrdU antibody.

### Whole-mount in situ hybridization and immunohistochemistry.

Whole-mount in situ hybridization of zebrafish embryos at 24 hpf and 72 hpf was performed by using digoxigenin labeled RNA probes as described previously [47]. Probes used: *nbc1* (NM_001034984), *trpM7* (NM_001030061), and *wt1a* (NM_131046). Stained embryos were photographed using Leitz MZ12 microscope and Spot Image digital camera. Whole-mount immunohistochemistry was performed as described in [19]. Anti-GFP, monoclonal antibody alpha6F ([48]; Developmental Studies Hybridoma Bank), anti-acetylated tubulin (6–11-B1; Sigma), and anti-BrdU (clone BU-133, Sigma) primary antibodies were used. For confocal imaging, Alexa-labeled secondary antibodies (Invitrogen) were used and imaged using a Zeiss LSM5 Pascal confocal microscope.

## Supporting Information

Figure S1 CD41:GFP Transgenics Express GFP in Multiciliated Cells of the Pronephros (A) 2-dpf CD41:GFP transgenic stained with anti-alpha6 NaK ATPase (red) and anti-GFP (green). (B) 7-dpf CD41:GFP transgenics stained with anti-acetylated tubulin (cilia, red) and anti-GFP (green).
(674 KB TIF)

Figure S2 Camptothecin Treatment Blocks Embryo Development and Cell Proliferation (A and B)The difference in BrdU incorporation in the tail region between a 1% DMSO control embryo (A) and an embryo incubated in 60 μM camptothecin (B).
(C and D) Camptothecin blocks early embryo development. 1% DMSO and 30 μM camptothecin were applied to dechorionated embryos at 3 hpf. The embryos were fixed at 7 hpf. While the control embryos progressed normally to about 60% epiboly, the camptothecin-treated embryos were completely arrested at the blastula stage.
(1.79 MB TIF)

Figure S3 Blockade of Fluid Flow Does Not Prevent Glomerular Fusion In situ hybridization with *wt1a* probe was used to localize podocytes in control 72-hpf (A) and obstructed 72-hpf (B) embryos (obstruction in (B) was initiated at 24 hpf). Arrows mark the area of midline fusion of podocytes in both (A) and (B).
(C) Light microscopic view of fused, cystic glomeruli in a 48-hpf obstructed embryo (obstruction was initiated at 24 hpf).
(5.56 MB TIF)

Figure S4 The ET11–9–Positive Segment Marks the Proximal-Most Extent of Multiciliated Cells Two-dpf ET11–9 embryos were stained with anti-acetylated tubulin (A; red) and anti-GFP (B; green). (C) Merge of (A) and (B). (D) Close-up of the anterior (proximal) end of the ET11–9 GFP-positive domain showing compressed, acetylated tubulin-positive bundles of cilia in the lumen of GFP-positive tubule cells. Dorsal acetylated tubulin-positive cells are neurons. Arrows mark the most proximal bundles of cilia originating from pronephric multiciliated cells [23].
(9.06 MB TIF)

Video S1 Proximal Cell Migration in ET33-D10 Transgenic Embryos The embryo is imaged between 2.5 dpf and 3 dpf. The pronephric epithelial cells are seen migrating in two nephrons. GFP-positive cells of the skin and the somite boundaries are stationary and serve as a frame of reference The head is on the left. Frame interval is 30 min. Number of frames is 19. Each frame is a projection of a confocal stack.
(1.35 MB MOV)

Video S2 Proximal Cell Migration in ET11–9 Transgenic Embryos The embryo is imaged at 2 dpf. The pronephric epithelial cells are seen migrating in two nephrons. The cells of the skin provide a stationary frame of reference. The head is on the left. Frame interval is 45 min. Number of frames is 11. Each frame is a projection of a confocal stack.
(962 KB MOV)

Video S3 Proximal Migration in CD41:GFP Transgenic Embryos The embryo is imaged between 2.5 dpf and 3 dpf. The pronephric GFP-positive multiciliated epithelial cells are seen migrating in two nephrons. The transient bright streaks are produced by circulating cells in the artery (above) and the vein (below). Between the artery and the vein, the GFP-positive hematopoietic progenitors are seen, some are disappearing into the circulation through the course of the recording. The first track of multiciliated cells is seen underneath the vein in this orientation of the fish (marked by the black arrowhead). The second track is seen deep, behind the venous circulating cells. The single dendritic-shaped cell in the skin sets the reference point. The head is on the left. Frame interval is 20 min. Number of frames is 25. Each frame is a projection of a confocal stack.
(1.88 MB MOV)

Video S4 Proximal Migration in NaK ATPase:GFP Transgenic Embryos The embryo is imaged between 2.5 and 3 dpf. The anterior pronephric epithelial cells are seen migrating in two nephrons. The migration is significantly slower in the most proximal nephron (left) where the tubule widens and cells pile up. Cell division can be also observed in this time lapse. The GFP-positive cells of the skin set the frame of reference. The head is on the left. Frame interval is 30 min. Number of frames is 30. Each frame is a projection of a confocal stack.
(1.48 MB MOV)

Video S5 Proximal Migration in *ret1*:GFP Transgenic Embryos The embryo is imaged between 1.5 dpf and 2 dpf. The pronephric epithelial cells are seen migrating in two nephrons. The cells near the cloaca and somite outlines set the frame of reference. The head is on the left. Frame interval is 30 min. Number of frames is 16. Each frame is a projection of a confocal stack.
(614 KB MOV)

Video S6 Proximal Pronephric Migration and Proximal Convolution in the NaK ATPase:GFP Transgenic Embryo The embryo is imaged between 2 dpf and 3 dpf. The pronephric epithelial cells are seen migrating in two nephrons. The migrating cells decelerate proximally and “pack' the proximal tubule as it forms a hairpin fold. Arrowheads highlight the areas of proximal tubule convolution. By 3 dpf, the gut tube expresses GFP and expands ventrally and posterior to the pronephric proximal tubules. The cells of the skin provide a stationary frame of reference. The head is on the left. Frame interval is 60 min. Number of frames is 22. Each frame is a flattened confocal stack.
(1.25 MB MOV)

Video S7 Close-Up of Proximal Convolution in a NaK ATPase:GFP Transgenic Embryo The proximal pronephros adjacent to the glomerulus (dashed circle; *gl*) is imaged for 28 h between 48 and 76 hpf. Large arrowheads mark the position of proximal convolution in both tubules; small arrows mark the migrating cells of the proximal nephron. Skin ionocytes and the gut epithelium are also fluorescent due to expression of GFP from the NaK ATPase a1.A4 subunit promoter. Frame interval is 30 min. Number of frames is 57. Each frame is a flattened confocal stack.
(1.36 MB MOV)

Video S8 Individual GFP-Positive Multiciliated Cell Migration Images of an individual cell from Video S3 (CD41:GFP transgenic) were rendered in grayscale and contrast enhanced to show the protrusive activity of the cell leading edge. This movies loops forward and backward to highlight the migratory cell behavior. Anterior is left. Frame interval is 20 min. Number of frames is 11. Each frame is a flattened confocal stack.
(782 KB MOV)

Video S9 Pronephric Epithelial Migration Is Independent of Cell Division An ET11–9 transgenic embryo was imaged between 2.5 and 3 dpf in the presence of 60 μM camptothecin. Pronephric epithelial cells are seen migrating in two nephrons. Anterior is to the left. Frame interval is 20 min. Number of frames is 22. Each frame is a projection of a confocal stack.
(702 KB MOV)

Video S10 The Rate of Proximal Migration Sharply Increases between 28 and 29 hpf The NaK ATPase:GFP transgenic embryo was imaged between 24 and 33 hpf. The arrow provides a point of reference. The color of the arrow changes to gray at 28.5 hpf. The GFP-positive cells of the skin set the frame of reference. The head is on the left. Frame interval is 30 min. Number of frames is 19. Each frame is a projection of a confocal stack.
(291 KB MOV)

Video S11 Pronephric Fluid Excretion prior to Glomerulus Formation A 30-hpf embryo was immobilized and the cloaca region was covered with petroleum jelly and immersed in mineral oil. The cloaca region was imaged every 60 s. Small fluid droplets appear under the cloaca and fuse with other droplets on the larval skin. Arrowheads mark the cloacal opening.
(1.75 MB MOV)

Video S12 Proximal Migration Is Abolished in an Obstructed NaK ATPase:GFP Transgenic Embryo Unilateral obstruction shown here resulted in a significant inhibition of proximal migration while the ability of the cell to move was preserved resulting in a random movement with virtually no proximal migration. In contrast, an unobstructed tubule shows normal proximal migration. The mechanical obstruction was performed at 1 dpf and the time lapse recording was performed between 2.5 and 3 dpf. The GFP-positive cells of the skin set the frame of reference. The head is on the left. Frame interval is 30 min. Number of frames is 20. Each frame is a projection of a confocal stack.
(839 KB MOV)

Video S13 Obstructed Kidneys Exhibit Circumferential Migratory Activity Mechanical obstruction was performed in this ET11–9 transgenic fish at 1 dpf and the time lapse recording was performed in this between 2.5 and 3 dpf. The head is on the left. Frame interval is 30 min. Number of frames is 24. Each frame is a projection of a confocal stack.
(1.44 MB MOV)

Video S14 Proximal Migration Is Blocked in *polycystin2* Morphant Kidneys and Cells Exhibit Circumferential Migratory Activity An ET33-D10 transgenic fish was injected with *polycystin2* exon12 splice donor morpholino. The time lapse recording was performed between 2.5 and 3 dpf. The head is on the left. Frame interval is 30 min. Number of frames is 20. Each frame is a projection of a confocal stack.
(1.28 MB MOV)

Video S15 Proximal Migration Is Blocked in *Ift88* (*polaris*) Morphant Kidney ET33-D10 transgenic fish were injected with *ift88* exon2 splice donor morpholino. The time lapse recording was performed between 2.5 and 3 dpf. The head is on the left. Frame interval is 30 min. Number of frames is 21. Each frame is a flattened confocal stack.
(523 KB MOV)

Video S16 Eliminating Glomerular Filtration Results in Marked Inhibition of Proximal Migration in Anterior (Proximal) Kidney An ET33-D10 transgenic fish was injected with cardiac *tnnt2* morpholino to eliminate cardiac contraction. The time lapse recording was performed between 2.5 and 3 dpf. The markedly reduced proximal migration was confined to the most distal portion of this segment (right). The head is on the left. Frame interval is 30 min. Number of frames is 16. Each frame is a projection of a confocal stack.
(735 KB MOV)

Video S17 Eliminating Glomerular Filtration Results in the Ectopic Kidney Convolution An ET11–9 transgenic embryo was injected with cardiac *tnnt2* morpholino to eliminate cardiac contraction. The time lapse recording was performed between 2.5 and 3 dpf. Proximal migration was normal in most of the segment except the most proximal portion, where a drop in the migration rate was accompanied by the development of convolution and the thickening of the tubule. Also note that transient obstruction developed and quickly resolved in one of the tubules. The head is on the left. Frame interval is 30min. Number of frames is 28. Each frame is a projection of a confocal stack.
(900 KB MOV)

## Abbreviations

dpf: days post-fertilization
hpf: hours post-fertilization
IFT: intraflagellar transport protein
phospho-FAK: phosphorylated Focal Adhesion Kinase
TRP: transient receptor potential
